# Both Physical Exercise and Progressive Muscle Relaxation Reduce the Facing-the-Viewer Bias in Biological Motion Perception

**DOI:** 10.1371/journal.pone.0099902

**Published:** 2014-07-02

**Authors:** Adam Heenan, Nikolaus F. Troje

**Affiliations:** 1 Queen’s University, Department of Psychology, Kingston, Ontario, Canada; 2 Queen’s University, School of Computing, Kingston, Ontario, Canada; 3 Queen’s University, Department of Biology, Kingston, Ontario, Canada; 4 Canadian Institute for Advanced Research, Toronto, Ontario, Canada; University of Münster, Germany

## Abstract

Biological motion stimuli, such as orthographically projected stick figure walkers, are ambiguous about their orientation in depth. The projection of a stick figure walker oriented towards the viewer, therefore, is the same as its projection when oriented away. Even though such figures are depth-ambiguous, however, observers tend to interpret them as facing towards them more often than facing away. Some have speculated that this facing-the-viewer bias may exist for sociobiological reasons: Mistaking another human as retreating when they are actually approaching could have more severe consequences than the opposite error. Implied in this hypothesis is that the facing-towards percept of biological motion stimuli is potentially more threatening. Measures of anxiety and the facing-the-viewer bias should therefore be related, as researchers have consistently found that anxious individuals display an attentional bias towards more threatening stimuli. The goal of this study was to assess whether physical exercise (Experiment 1) or an anxiety induction/reduction task (Experiment 2) would significantly affect facing-the-viewer biases. We hypothesized that both physical exercise and progressive muscle relaxation would decrease facing-the-viewer biases for full stick figure walkers, but not for bottom- or top-half-only human stimuli, as these carry less sociobiological relevance. On the other hand, we expected that the anxiety induction task (Experiment 2) would increase facing-the-viewer biases for full stick figure walkers only. In both experiments, participants completed anxiety questionnaires, exercised on a treadmill (Experiment 1) or performed an anxiety induction/reduction task (Experiment 2), and then immediately completed a perceptual task that allowed us to assess their facing-the-viewer bias. As hypothesized, we found that physical exercise and progressive muscle relaxation reduced facing-the-viewer biases for full stick figure walkers only. Our results provide further support that the facing-the-viewer bias for biological motion stimuli is related to the sociobiological relevance of such stimuli.

## Introduction

Biological motion stimuli, depicted as orthographically projected stick figure walkers, are depth-ambiguous. Any facing orientation for such a stimulus therefore provides observers with two veridical percepts. For example, the fronto-parallel projection of a stick figure walker displayed facing the viewer is the same as the projection of it facing away. Although the information contained in the image supports both percepts equally, naïve observers perceive these stimuli as facing towards them more often than facing away. This facing-the-viewer bias has been found repeatedly [1]–[5], and researchers have identified both top-down (e.g., walker gender [1], [2]) and bottom-up factors (e.g., kinematic and structural motion; [6]) that contribute to it.

Since the facing-the-viewer bias appears to be specific to human-like stimuli, some have suggested that it may exist for sociobiological reasons [2], [4]. That is, a bias to perceive human figures with ambiguous orientations as facing towards oneself would increase the likelihood that one would be ready to respond adequately. This hypothesis posits that humans developed the facing-the-viewer bias because mistaking an approaching person as receding is potentially more costly than the opposite error. In support of this hypothesis, there is evidence that observers perceive male walkers as facing towards them more often than female walkers [1], [2], which is significant because males are typically perceived as more threatening than females [7]. Furthermore, while upright biological motion stimuli elicit significant facing-the-viewer biases, inverted ones do not [4]. This is important, as naïve observers do not readily identify inverted biological stimuli as human [8]–[10].

The theory that the facing-the-viewer bias exists for sociobiological reasons implies that observers may perceive the facing-towards percept of biological motion stimuli as more threatening. Measures of anxiety and the facing-the-viewer bias should therefore be related, as researchers have consistently found that anxious individuals display an attentional bias towards more threatening stimuli [11]–[13]. With respect to ambiguous visual stimuli, more anxious individuals display a bias towards perceiving the more threatening percept compared to those who are less anxious [14]–[16]. For example, more anxious individuals perceived faces expressing anger or fear more often than positive ones at stimulus onset than less anxious people on a binocular rivalry task (i.e., a task that elicits perceptual alternations by presenting a different stimulus to each retina simultaneously; [15]). The same was found for individuals diagnosed with social anxiety disorder or panic disorder compared to healthy controls [16]. In fact, some argue that this threat bias may contribute to and maintain anxiety disorders, as individuals with such disorders are unconsciously biased to attend to stimuli that in turn evoke more anxiety in a feed-forward loop [11], [17]–[19].

While there has been relatively little investigation into how anxiety relates to the facing-the-viewer bias thus far, it has been shown that participants with greater attachment anxiety had greater facing-the-viewer biases than less anxious people [20]. On the other hand, there is evidence that a group of individuals who scored high on a measure of social anxiety (i.e., more anxious) had weaker facing-the-viewer biases than a group who scored low on the same measure [21]. Both the aforementioned studies were correlational, and to date, there have been no studies in which anxiety has been manipulated (directly or otherwise) in order to examine effects on facing-the-viewer biases.

## Experiment 1

One method of indirectly reducing anxiety that has received much support is engaging in physical exercise [22]–[27]. The exact mechanisms by which this occurs, however, remain underspecified. One possibility is that exercise provides people with an explanation for their physiological arousal, thus preventing them from attributing heightened arousal as related to external stimulation [23], [25]. Such a hypothesis would explain why physical exercise has proven a successful treatment for specific phobias [25], [28]. Regardless of the underlying mechanism, physical exercise provides a tool by which we can experimentally reduce participants’ anxiety indirectly in order to assess any corresponding changes in facing-the-viewer biases.

The purpose of Experiment 1 was to investigate the relationship between anxiety and the facing-the-viewer bias by assessing participants’ biases after their anxiety was reduced (indirectly) via engaging in physical exercise. Participants completed anxiety questionnaires, exercised on a treadmill, and then immediately completed a perceptual task that allowed us to assess their facing-the-viewer bias. To avoid confounding the variable of interest with a simple response bias (e.g., Is the walker facing towards or facing away?), we presented stick figure walkers rotating about a vertical axis and asked participants to indicate their spinning direction. Together with information about the “veridical” orientation of the 3D walker, we inferred perceived facing direction from participants’ responses [29].

We randomly assigned participants to three physical exercise conditions on the treadmill: Standing (0 km/h), walking (4 km/h), or jogging (8 km/h). As anxiety has been found to correlate positively with the facing-the-viewer bias [20] and the anxiolytic benefits of physical exercise have been consistently reported [22]–[27], we hypothesized that facing-the-viewer biases would be lower for participants who exercised (walking or jogging) than for participants who were merely standing. Furthermore, we assessed facing-the-viewer biases for three types of biological motion stimuli: Full stick figure walkers, bottom-half-only, and top-half-only. We hypothesized that exercise would weaken the facing-the-viewer bias for full walkers, but that this would not happen (or would happen to a smaller degree) if we showed only parts of the body. Lastly, we hypothesized that anxiety assessed prior to participants physically exercising would positively correlate with the observed facing-the-viewer bias and that this would be particularly visible in the standing condition. In the walking or jogging conditions, we expected participants to experience reductions in anxiety relative to pre-exercise levels and that this would reduce or even eliminate correlations between anxiety and the facing-the-viewer bias.

## Methods

### Ethics Statement

The Queen’s University General Research Ethics Board (GREB) approved this research and all methods were in accordance with the Declaration of Helsinki. Upon arriving at the lab, each participant provided verbal and written informed consent before completing any questionnaires. At the end of the experiment, participants were verbally debriefed and given a written debriefing form complete with contact information for the Queen’s University GREB. In line with the Queen’s University GREB and Canadian federal law, we did not require parental consent from participants who were under the age of 18 at the time of their participation in this study, as post-secondary students are considered able to provide their own consent in Canada. The Queen’s University GREB approved this consent procedure.

### Participants

Sixty-six naïve undergraduate and graduate students participated in this experiment (42 women, 24 men). All participants had normal or corrected-to-normal vision and had never participated in any experiment that involved biological motion stimuli. Participants ranged in age from 17 to 26 (*M* = 19.82 years, *SD* = 2.29 years). We recruited participants either through an undergraduate participant pool or a voluntary participant pool comprised of both undergraduate and graduate students. Participants recruited from the voluntary participant pool received $10.00 (CAD) for participation. Participants recruited from the undergraduate participant pool received partial course credit. Of these 66 participants, 11 were excluded (10 women, 1 man) for either failing to respond correctly to enough control trials (minimum 75% accuracy required; *n* = 10; all women) or because they were a statistical outlier (*z* score>3) in terms of their facing-the-viewer bias scores (*n* = 1; a man). All statistical analyses were performed on the remaining 55 participants (32 women, 23 men), who ranged in age from 18 to 26 (*M* = 19.95 years, *SD* = 2.32 years).

### Stimuli and Apparatus

Stimuli consisted of depictions of solid cubes and stick figure walkers. The cubes were tilted 10° degrees with respect to the horizontal plane such that the camera looked at them slightly from above. They were opaque and thus unambiguous. The 3D cubes rotated at a speed of 45°/s about a vertical axis, half of them clockwise and the other half counterclockwise. They were rendered with an orthographic camera and measured 5 cm on-screen, subtending a visual angle of 4.75° for the observer.

**Figure 1:**
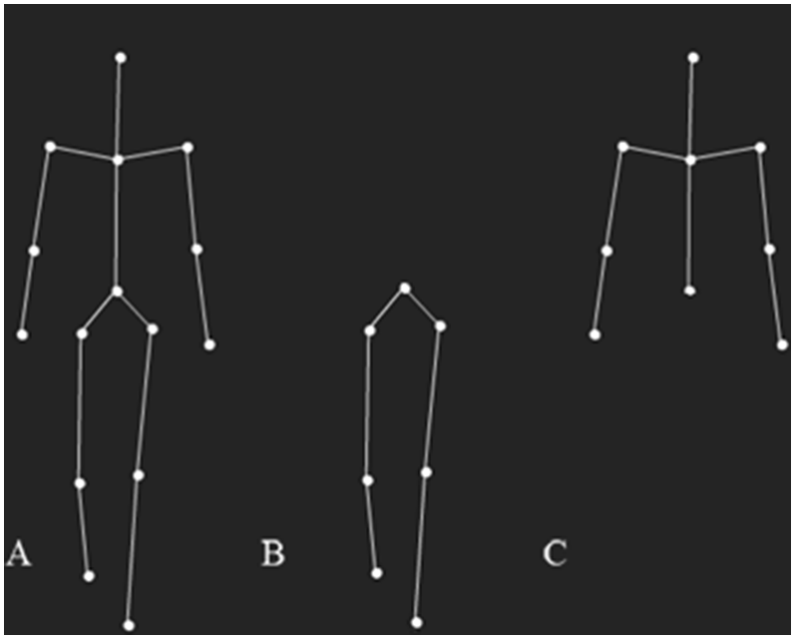
Example stimuli from the perceptual task used in both Experiments 1 and 2 are displayed. Stimuli included three types: (A) Full stick figure walkers, (B) bottom-half-only stick figure walkers, and (C) top-half-only stick figure walkers. Stick figure walkers were based on 3D biological motion point-light walkers consisting of 15 dots (depicting the center of major skeletal joints; see Troje, 2002, 2008) to which we added connecting lines.

Stick figure walkers were based on 3D biological motion point-light walkers, consisting of 15 dots depicting the center of major skeletal joints [30], [31] to which we added connecting lines. We used three versions of this stimulus: Full stick figure walkers, bottom-half-only, and top-half-only (see Figure 1). The initial phase of each walker (i.e., the posture of the walker at stimulus onset) was varied at random. Stick figures were rendered using an orthographic camera with a horizontal optical axis and appeared white on a black background. The 3D stick figures rotated counterclockwise at a speed of 45°/s about their vertical axis. The initial camera azimuth (i.e., the horizontal viewpoint) covered the whole range from 0° (frontal view) to 360° in 30° increments. Full walkers were 7.5 cm high on the screen, subtending a visual angle of 7° for the observer. Bottom-half-only walkers were 4 cm high, subtending a visual angle of 3.75°, while top-half-only walkers were 3.5 cm high, subtending a visual angle of 3.25° (see Video S1).

The treadmill used in this experiment had a deck measuring 50 cm wide by 146 cm long and had an electronic display that showed time and speed (Advanced Fitness Group, Cottage Grove, Wisconsin). Participants were not allowed to alter their speed while on the treadmill, but they were allowed to discontinue at any time should they experience discomfort (no participants discontinued).

### Questionnaires

Participants completed both the State and Trait forms of the State-Trait Anxiety Inventory (STAI; [32]), which consists of 40 items and is designed to assess how anxious an individual is currently feeling (i.e., state anxiety) and how anxious an individual typically tends to feel (i.e., trait anxiety). Participants also completed the Social Interaction Anxiety Scale (SIAS; [33]), which is a questionnaire that consists of 20 items designed to assess how anxious an individual feels in situations involving social interaction (e.g., at a dinner party, meeting new people). Participants also completed a simple demographic questionnaire (e.g., age, gender). We hypothesized that facing-the-viewer biases would correlate most strongly with SIAS scores, given that the facing-the-viewer bias is thought to be socially relevant and that the SIAS is specifically designed to measure social anxiety.

### Design

We used a mixed factorial design with two factors, one between-subject factor (Exercise) and one within-subject factor (Stimulus). Each factor had three levels. For Exercise, we randomly assigned each participant to a treadmill speed: 0 km/h (standing/control condition), 4 km/h (walking condition), or 8 km/h (jogging condition). For Stimulus, we presented each participant with three different types of stimuli: Full stick figure walkers, bottom-half-only, and top-half-only.

In total, each participant observed 144 stick figure trials (12 initial camera azimuths x 3 stimulus types x 4 repetitions) and 12 solid cube trials (same cube presented from 12 camera azimuths). Note, that even though all 3D stick figures rotated counterclockwise, we still covered all possible 2D stimuli. For instance, a clockwise rotating stick figure with an initial camera azimuth of 30° rendered with an orthographic camera would have resulted in exactly the same 2D stimulus as the projection of a stick figure rotating counterclockwise with an initial camera azimuth of 150°. Solid cubes were dispersed equally throughout the trials such that a cube would be presented every 12 trials. These unambiguous cubes were presented as a control to ensure participants understood the concepts of clockwise and counterclockwise rotation and were not responding randomly.

### Procedure

At the time of booking appointments, the experimenter asked participants to wear attire that was appropriate for exercise and instructed them that wearing running shoes was mandatory. A reminder email was sent out the night before participants were scheduled to participate in order to emphasize the importance of appropriate attire.

Upon arriving at the lab, each participant provided verbal and written informed consent before completing the STAI, SIAS, and demographic questionnaires. After completing the questionnaires, the experimenter led the participant to the treadmill and measured the participant’s heart rate by asking participants to count the number of beats (measured by placing a finger on the carotid artery in their neck) for 15 s. Subsequently, the experimenter informed participants of the condition that they had been randomly assigned to and set the speed of the treadmill to either 0 km/h (standing/control condition), 4 km/h (walking condition), or 8 km/h (jogging condition). Participants then remained on the treadmill for 10 min. During that time, they were not allowed to use their cell phones or perform any other tasks.

After completing the treadmill exercise, the experimenter measured the participant’s heart rate again and then participants immediately performed the perceptual task. Without informing participants of the ambiguous nature of the stimuli, the experimenter instructed the participant to press the ‘S’ key if the stick figure or the cube rotated clockwise, or the ‘K’ key if they rotated counterclockwise. Participants first completed a 12-trial practice block to familiarize themselves with the task, and also to familiarize themselves with judging clockwise and counterclockwise. The experimenter asked the participant to complete the practice block again if the participant did not understand the task. The 156 trials of the main experiment were then presented.

Both walker and cube stimuli were shown for 0.5 s. Following each stimulus presentation, observers were given a 2 s window to respond as to whether they saw the figure rotating clockwise or counterclockwise. Then the next trial began. If observers had not responded within this window, the trial was discarded from the analysis.

The experiment took place in a dimly lit room. Participants sat at a desk with a desktop computer in front of them consisting of a mouse, keyboard, and a 17″ cathode ray tube screen running at 100 fps. Observers sat approximately 60 cm from the screen and kept their eyes level with the screen by adjusting the chair once before starting the experiment.

Upon completing the task, participants were verbally debriefed and also given a debriefing form. Overall, testing lasted approximately 30 min.

### Data Analyses

Our measure for the facing-the-viewer bias was based on modelling the collected observer responses in terms of a generalized linear model (GLM):
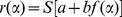
(1)Here, *r* is the rate of counterclockwise responses. α expresses camera azimuth in degrees. *S* is the logistic function: *f* is a rectangular function which adopts a value of 1 if −90°<α<90°, −1 if 90°<α<270°, and 0.5 if α is either −90° or 90°. *S* is the logistic function: *S*(x) = 1/(1+e^−x^). The variables *a* and *b* are the predictors of the model. The first predictor *a* accounts for a general rotation direction bias, that is, for a preference for responding “clockwise” (if *a*<0) or “counterclockwise” (if *a*>0). The second predictor, *b*, accounts for the degree to which the dependency of participants’ responses on horizontal viewpoint (camera azimuth, α) follows the expected influence of camera azimuth on perceived rotation direction, *f*(α). The variable *b* is therefore a measure of the facing-the-viewer bias and constitutes our main dependent variable, which is henceforth referred to as “FTV score”.

Note that parameters *a* and *b* of the GLM do not have easily interpretable units. Without any modification by the other effect (i.e. if *b* = 0), a rotation direction bias of *a* = 1 would correspond to a response rate of 73% “counterclockwise” responses, an effect of *a* = 0 would correspond to a response rate of 50%, and an effect of *a* = −1 would correspond to a response rate of 27% “counterclockwise”. Likewise, given that *a* = 0, a FTV score of *b* = 1 would indicate that participants perceived the walker facing towards them in 73% of the cases, whereas *b* = −1 would indicate that they would see the walker only in 27% of the cases facing towards them and facing away in the remaining 73%.

We used 3 (Exercise) x 3 (Stimulus) mixed design ANOVAs to analyze FTV scores and reaction times. As a note regarding our analysis of FTV scores, we did not examine the stability of FTV scores over time as we have found previously (e.g., [20]) that this bias is relatively stable. For repeated-measures variables (i.e., Stimulus and the Exercise x Stimulus interaction), Wilk’s λ was used whenever the assumption of sphericity was violated. For all post-hoc comparisons, inflation of family-wise error rate was controlled using a Bonferroni correction. We also performed correlations. For these, we corrected for inflating false discovery rate by employing the correction method described first by Simes [34], and then updated by Benjamini and Hochberg [35]. For this correction method, we set the chance of making a false discovery at *Q* = .05 and adjusted corresponding *p* values for correlation coefficients according to the formula *p*_adjusted_ = (*c*/*k*)(*p*_unadjusted_)_,_ where *c* represents the number of comparisons, and *k* was determined by the rank after sorting original *p* values by magnitude (see [35]). All significant correlations described below are significant after performing this correction unless otherwise specified and all *p*_adjusted_ statistics are compared with α = .05 to judge statistical significance.

## Results

### Sample Statistics

Participants’ mean state anxiety score from the STAI was 31.67 (*SD* = 7.53), mean trait anxiety score from the STAI was 35.85 (*SD* = 8.46), and mean social interaction anxiety score from the SIAS was 20.04 (*SD* = 10.20). All three outcomes are typical of nonclinical populations and indicate normal levels of anxiety symptoms. To examine whether there were any gender differences in our data, we used individual sample *t* tests to compare men and women. Men and women did not differ significantly in terms of age, heart rate (before or after exercise), any anxiety measure, or FTV scores for any of the levels of factor Stimulus (all *p*’s>.05).

### Number of Missed Responses

For the perceptual task data, a 3 (Exercise) x 3 (Stimulus) mixed design ANOVA on the number of missed trials (i.e., no response within 2 s of previous stimulus presentation) revealed no effect of Exercise (*F*<1), no effect of Stimulus (*F* (2, 104)  = 1.35, *p* = .265, η^2^_partial_ = .02), and no significant interaction (*F*<1). On average, participants missed 3.90% (i.e., about 5 trials) of the walker stimuli (*SD* = 4.75%).

### Heart Rate

We performed a one-way ANOVA on the difference between pre- and post-exercise heart rates across levels of Exercise. Heart rates were measured in beats per minute (bpm). The ANOVA was significant, *F* (2, 52) = 60.49, *p*<.001, η^2^_partial_ = .70, with participants having significantly greater heart rate increases (relative to pre-exercise baseline) in the jogging condition (*M* = 56.10 bpm increase, *SD* = 20.44) compared to in the standing (control) condition (*M* = 3.47 bpm increase, *SD* = 7.23, *p*<.001). Participants’ heart rates increased somewhat more in the walking condition (*M* = 12.00 bpm increase, *SD* = 14.42) compared to the control condition, but the difference between the two conditions did not reach significance (*p* = .325).

### FTV Scores

We analyzed effects on FTV scores with a 3 (Exercise) ×3 (Stimulus) mixed design ANOVA. We found a significant main effect of Stimulus, Wilk’s λ = .49, *F* (2, 104) = 26.16, *p*<.001, η^2^_partial_ = .51, as well as a significant Exercise x Stimulus interaction, Wilk’s λ  = .76, *F* (4, 104)  = 3.83, *p* = .006, η^2^_partial_ = .13. The main effect of Exercise was not significant, *F* (2, 52) = 1.82, *p* = .172, η^2^_partial_ = .07.

**Figure 2:**
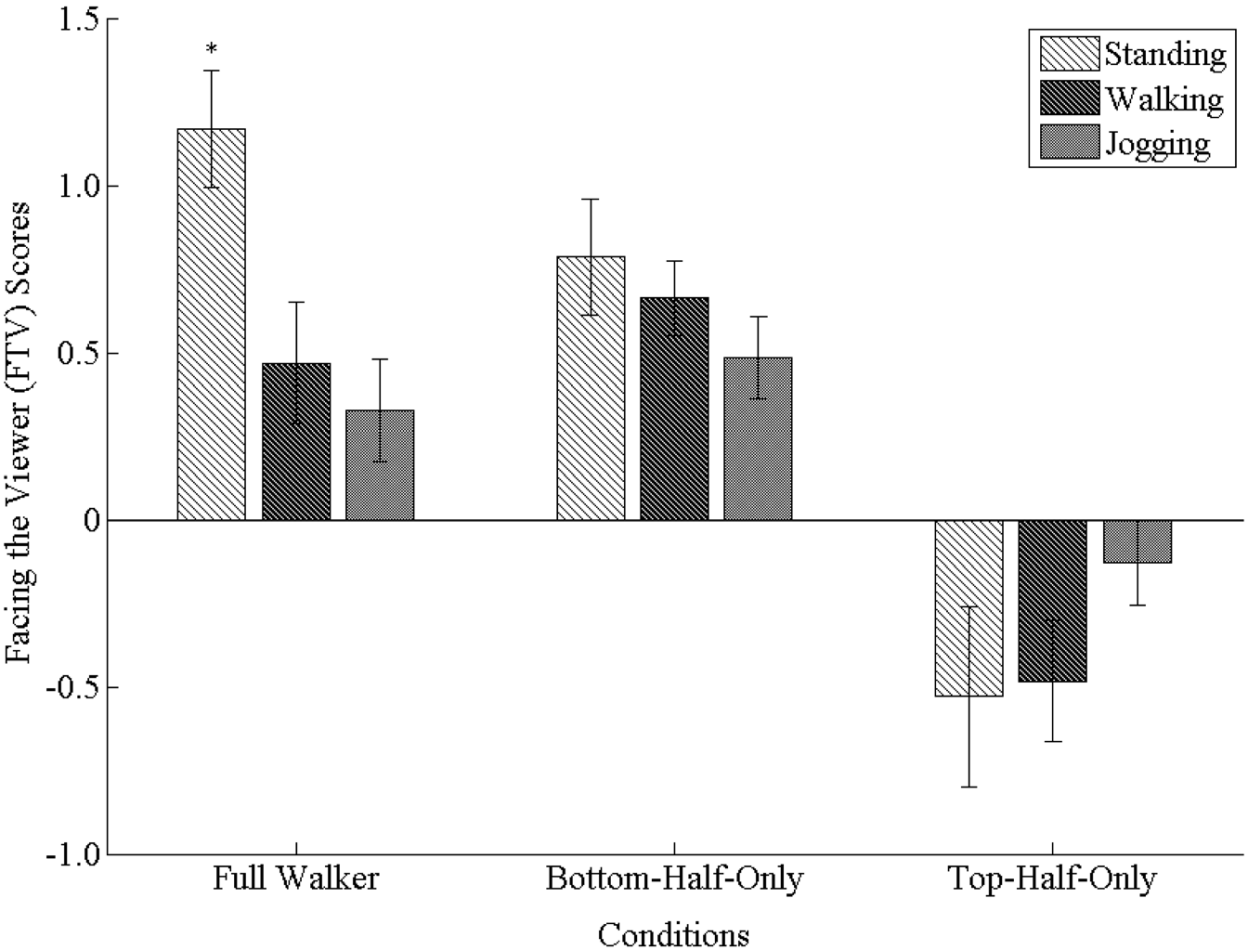
Mean facing-the-viewer (FTV) scores for full stick figure walkers, bottom-half-only stick figure walkers, and top-half-only stick figure walkers (grouped on x-axis) in Experiment 1. Mean FTV scores are displayed separately for the standing, walking, and jogging exercise conditions (grouped as differently shaded bars). There was a significant interaction between Exercise condition and Stimulus type. Note that *indicates a significant simple main effect, whereby FTV scores in the standing condition were significantly greater than either the walking or jogging conditions for full stick figure walkers only. No other simple main effects were significant. Error bars represent standard error of the mean.

For the significant main effect of Stimulus, post-hoc comparisons revealed that FTV scores for full and bottom-half-only stick figure walkers were significantly greater than FTV scores for top-half-only walkers (for both comparisons, *p*<.001). Full and bottom-half-only stick figure walkers, however, did not differ significantly from each other. To further examine these biases, we performed one-sample *t* tests on FTV scores separately for each level of Stimulus (collapsed across all levels of Exercise) against *b* = 0 (i.e., no bias). These *t* tests revealed that both full and bottom-half-only stick figures elicited significant facing-the-viewer biases, while top-half-only stimuli elicited a significant facing away bias (i.e., a negative FTV score; all cases *p*<.001; see Figure 2).

To analyze the significant interaction between Exercise and Stimulus, we performed a simple main effects analysis. As hypothesized, participants in the walking (*p* = .021) and jogging conditions (*p* = .005) had significantly lower FTV scores than in the standing condition for full stick figure walkers only (see Figure 2). On the other hand, participants in the jogging condition did not differ significantly from those in the walking condition in terms of FTV scores for full walkers. Although it appears as if FTV scores for top-half-only stimuli might differ between Exercise condition if we had more statistical power, differences in FTV scores between Exercise conditions for either bottom-half-only or top-half-only stimuli did not approach statistical significance.

We also analyzed the simple main effects of Stimulus separately for each level Exercise. For the standing condition, FTV scores for top-half-only stimuli were significantly lower than for either full (*p*<.001) or bottom-half-only stimuli (*p*<.001), but full and bottom-half-only walkers did not differ significantly from each other (*p* = .107; see Figure 2). For the walking condition, we observed the same pattern: FTV scores for top-half-only stimuli were significantly lower than for either full stick figure walkers (*p*<.001) or bottom-half-only stimuli (*p*<.001), but full and bottom-half-only stimuli did not differ significantly (*p* = .606). On the other hand, we observed no significant differences in FTV scores between Stimulus types for the jogging condition (all *p*’s>.05).

We then performed nine separate one-sample *t* tests for each combination of our two factors in order to compare FTV scores with zero (i.e., no bias). For the standing condition, we found that while the full and bottom-half-only stick figure walkers elicited significant facing-the-viewer biases (both *p*<.001), FTV scores for top-half-only stimuli did not differ significantly from zero (*p* = .070; see Figure 2). For the walking condition, we found significant facing-the-viewer biases for both full (*p* = .019) and bottom-half-only (*p*<.001) Stimulus types, and a significant facing away bias for top-half-only stimuli (*p* = .016). For the jogging condition, we once again observed significant facing-the-viewer biases for full (*p* = .046) and bottom-half-only stimuli (*p* = .001), while FTV scores for top-half-only stimuli did not differ significantly from zero (*p* = .327).

### Reaction Time

We examined differences in reaction times on the perceptual task using a 3 (Exercise) ×3 (Stimulus) mixed design ANOVA. Neither the main effect of Stimulus, Wilk’s λ  = .92, *F*(2, 104) = 2.10, *p* = .133, η^2^_partial_ = .08, or Exercise, *F* (2, 52)  = 0.59, *p* = .558, η^2^_partial_ = .02, was significant, nor was there a significant Exercise x Stimulus interaction, Wilk’s λ  = .91, *F*(4, 104) = 1.26, *p* = .290, η^2^_partial_ = .05. Reaction times, therefore, did not differ as a function of Exercise or Stimulus. The means and standard errors (in parentheses) were as follows: Full stick figure walkers, 0.72 s (0.04), bottom-half-only stimuli, 0.72 s (0.04), and top-half-only stimuli, 0.74 s (0.04).

### Correlations

#### FTV Scores and Anxiety Measures

**Figure 3:**
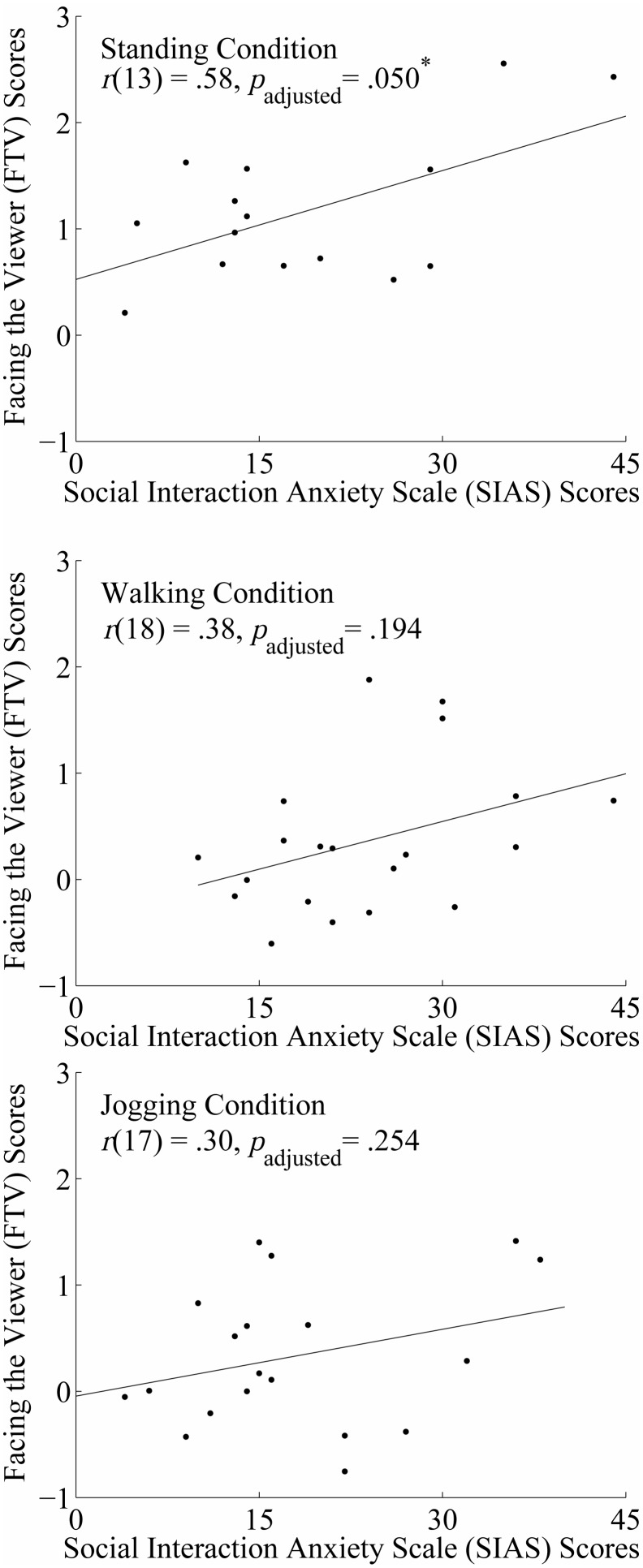
Scatterplots displaying Pearson’s r correlations between facing-the-viewer (FTV) scores (for full stick figure walkers) and participants’ scores on the social interaction anxiety scale (SIAS) in Experiment 1. Separate scatterplots are displayed for each level of Exercise condition, with the standing condition shown in the top figure, the walking condition shown in the middle, and the jogging condition shown on the bottom. Note that p values have been adjusted for the number of multiple comparisons within each condition.

We analysed correlations between anxiety (3 measures: STAI-state, STAI-trait, and SIAS) and FTV scores for full stick figure walkers separately for each level of Exercise, for a total of 18 comparisons (i.e., 6 comparisons per level of Exercise). One participant was excluded from correlational analyses (a male in the walking condition) as his data was a clear outlier after visually analyzing scatterplots due to a very large FTV score. Using the very conservative multiple comparison correction method described above, we found that anxiety measures did not significantly correlate with FTV scores in any of the three Exercise conditions (all *p*_adjusted_>.05). When we used a less conservative correction (i.e., corrected for multiple comparisons separately for each condition, or 6 comparisons), we found that SIAS scores and FTV scores for full stick figure walkers in the standing condition were significantly correlated, *r*(13) = .58, *p*_adjusted_ = .050. Specifically, individuals with greater social interaction anxiety had greater FTV scores in the standing condition (see Figure 3). This correlation was not significant, however, for either the walking, *r*(18) = .38, *p*_adjusted_ = .194, or jogging conditions, *r*(17) = .30, *p*_adjusted_ = .254. There were no other significant correlations between anxiety measures and FTV scores.

#### FTV Scores and Heart Rates

In accordance with the significant results of the ANOVA described above for FTV scores, we found a significant negative correlation between participants’ FTV scores for full stick figure walkers and participants’ heart rate measured immediately after they completed the treadmill task, *r*(53) = −.30, *p* = .027. Lower FTV scores for full stick figure walkers were associated with greater heart rates recorded after participants completed the exercise portion of the study.

#### FTV Scores and Reaction Times

Although the 3 (Exercise) ×3 (Stimulus) ANOVA on reaction times on the perceptual task indicated no significant differences, we examined the correlations between reaction times and FTV scores. For full stick figure walkers, there were no significant correlations between reaction times and FTV scores for any of the three levels of Stimulus. However, we did observe that reaction times correlated positively with FTV scores for bottom-half-only stimuli, and correlated negatively with FTV scores for top-half-only stimuli (see Table S1 for all correlation coefficients and inferential statistics). This means that biases (facing-towards for the bottom-half-only stimuli and facing-away for the top-half-only stimuli) became stronger the longer participants took to respond. Note that stimulus presentation time was always the same and that responses were only given after the stimulus had disappeared.

## Experiment 2

In Experiment 1, physical exercise reduced the facing-the-viewer bias for full stick figure walkers. We had originally hypothesized that this would be the case presumably because of the anxiolytic effects of exercise. This argument, however, is indirect. That is, it is possible that facing-the-viewer biases decreased after engaging in physical exercise due to the physiological effects of exercise (e.g., differences in oxygenation, vascular functioning). A more direct method of assessing whether anxiety relates to the facing-the-viewer bias is to attempt to manipulate anxiety directly and then observe the subsequent effect on the facing-the-viewer bias.

Inducing anxiety by asking participants to read scripts and to imagine personal experiences during which they experienced stress has been successful in increasing subjective anxiety in laboratory experiments [36]–[38]. Kimbrell et al., (1999), for example, found that participants who recalled stressful life events while looking at human faces expressing anxiety experienced significant increases in self-reported anxiety, as well as concomitant differences in brain activation (compared to controls) as measured by positron emission tomography (PET). There has also been one experiment in which facing-the-viewer biases were significantly affected by imagery scripts, as Heenan et al. (2012) found that reading a loneliness induction script significantly affected the relationship between attachment anxiety and the facing-the-viewer bias. Given the success that such imagery scripts have had in both increasing anxiety and affecting facing-the-viewer biases, we chose to adopt a similar imagery script in order to induce anxiety in Experiment 2.

We were also interested in experimentally reducing anxiety in Experiment 2. Progressive muscle relaxation is a method of reducing anxiety that involves flexing and releasing muscle groups throughout the body while focussing on the internal sensation of relaxation that this provides [39], [40]. A clinical study involving patients with cancer found that this relaxation technique was as effective as Alprazolam (a benzodiazepine) at significantly reducing symptoms of anxiety [41]. Progressive muscle relaxation has also been found to be an effective treatment for individuals with panic disorder [42], and is often used in conjunction with cognitive behavioural therapy to treat other anxiety disorders as well, such as specific phobias or obsessive-compulsive disorder [40].

The purpose of Experiment 2 was to investigate the relationship between anxiety and the facing-the-viewer bias by assessing participants’ biases after they engaged in a task designed to manipulate their level of anxiety. For the most part, the methodology of Experiment 2 was exactly the same as that of Experiment 1, except for the substitution of the exercise task with an anxiety manipulation task. Participants completed anxiety questionnaires, performed the anxiety manipulation task, and then immediately completed a perceptual task that allowed us to assess their facing-the-viewer bias. In order to reduce response bias, we presented rotating stick figure walkers and deduced the perceived facing orientation of walkers in the same way as we did in Experiment 1.

We randomly assigned participants to three conditions: A control condition that involved performing an innocuous word search task, an anxiety induction condition that consisted of a stressful imagery writing task, or an anxiety reduction condition that consisted of performing progressive muscle relaxation. As anxiety has been found to correlate positively with the facing-the-viewer bias [20], we hypothesized that participants in the anxiety induction condition would have greater facing-the-viewer biases than those in the control condition, while those in the relaxation condition would have lower facing-the-viewer biases than those in the control condition. As we did in Experiment 1, we assessed facing-the-viewer biases for three types of biological motion stimuli: Full stick figure walkers, bottom-half-only, and top-half-only. We hypothesized that anxiety induction and relaxation would increase and decrease (respectively) the facing-the-viewer bias for full walkers, but that this would not happen (or would happen to a smaller degree) for bottom-half- and top-half-only stimuli. Lastly, we added an additional anxiety measure in response to a methodological error in Experiment 1, whereby we did not directly assess anxiety immediately after participants completed the exercise task (i.e., a manipulation check). In Experiment 2, we assessed anxiety both before and after the anxiety manipulation (using a visual analogue scale). We hypothesized that anxiety assessed just after participants completed the anxiety manipulation would positively correlate with the facing-the-viewer bias.

## Methods

### Ethics Statement

Same as Experiment 1.

### Participants

Sixty-four naïve undergraduate students participated in this experiment (48 women, 16 men). All participants had normal or corrected-to-normal vision and had never participated in any experiment that involved biological motion stimuli. Participants ranged in age from 17 to 21 (*M* = 18.39 years, *SD* = 0.79 years). We recruited participants through an undergraduate participant pool. Participants received course credit (1%) towards the completion of a first year undergraduate psychology course. Of these 64 participants, 7 were excluded (6 women, 1 man) for failing to respond correctly to enough control trials (minimum 75% accuracy required). An additional two women were excluded because they were statistical outliers (*z* score>3) in terms of their anxiety scores. All statistical analyses were performed on the remaining 55 participants (40 women, 15 men), who ranged in age from 17 to 21 (*M* = 18.44 years, *SD* = 0.83 years).

#### Stimuli and Apparatus

All stimuli for the perceptual task were the same as those used in Experiment 1.

For the control condition, participants completed word searches. We downloaded word searches from a free website (http://www.puzzles.ca/wordsearch.html) and used the same 10 for each participant (no participant completed all 10 during the allotted time). These word searches were chosen because of their neutral word categories (e.g., song names, types of food). There were also written instructions describing how to complete the word searches.

For the relaxation condition, the experimenter guided the participant through a progressive muscle relaxation exercise. This task involves focusing one’s attention on different muscle groups of the body (e.g., feet, calves, shoulders) and then tensing and relaxing them. The experimenter was trained to perform this technique by a senior graduate student in clinical psychology. The procedure followed the script outlined by Bourne [40], that is based on the relaxation technique described first by Benson [39].

For the anxiety induction condition, participants were given a script that we created with the following instructions*: Please recall and write down a time when you felt very stressed or anxious. You will have 10 minutes to complete this. Be sure to write down the story in as much detail as you need in order to vividly remember and imagine this time in your life.* Participants were also told that they were to keep their stories when they were finished and to take these with them when they left the laboratory. If participants finished within the allotted time, they were instructed to think of another time and write about that until the experimenter told them the task was finished.

All participants completed a positive mood induction task at the end of the experiment. This task was identical to the anxiety induction task except that participants were instructed to write about a time when they felt very good about themselves (as opposed to very stressed). This task was included in order to ensure that any lingering negative or anxious mood was alleviated before participants left the experiment.

### Questionnaires

Participants completed the same questionnaires as they did in Experiment 1 (i.e., both the state and trait forms of the STAI, as well as the SIAS). Participants also completed a simple demographic questionnaire (e.g., age, gender), which was identical to that used in Experiment 1.

For the manipulation check, we assessed participants’ subjective anxiety using a visual analogue scale. Participants marked their responses by drawing a mark on a line that spanned from *No Anxiety* (far left) to *Most Anxiety You Have Ever Felt* (far right). The length of the line was 9 cm.

### Design

As in Experiment 1, we used a mixed factorial design with two factors, one between-subject factor (Condition) and one within-subject factor (Stimulus). Each factor had three levels. For Condition, we randomly assigned each participant to either the control condition (i.e., innocuous word searches), the anxiety induction condition (i.e., anxiety imagery script), or the relaxation condition (i.e., progressive muscle relaxation). For Stimulus, we presented each participant with three different types of stimuli: Full stick figure walkers, bottom-half-only, and top-half-only.

With respect to the perceptual task, the design, was exactly the same as it was in Experiment 1.

#### Procedure

Upon arriving at the lab, each participant provided verbal and written informed consent before completing the STAI, SIAS, and demographic questionnaires. After completing the questionnaires, the experimenter measured the participant’s heart rate by asking them to count the number of beats (measured by placing a finger on the carotid artery in their neck) for 15 s. The experimenter then asked participants to complete the visual analogue anxiety measure, followed by the anxiety manipulation part of the experiment. During this time, they were not allowed to use their cell phones or perform any other tasks. Participants in the control condition completed word searches after receiving both written and verbal instructions. Participants in the anxiety induction task performed the anxiety imagery writing task after receiving both written and verbal instructions. Participants in the relaxation condition were guided through the progressive muscle relaxation task by the experimenter. In all three conditions, participants completed these tasks for 10 minutes.

After completing the anxiety manipulation portion of the experiment, the experimenter measured the participant’s heart rate again and asked them to complete the visual analogue anxiety measure once more (participants were not allowed to see their previous response). Participants then immediately performed the perceptual task (see Experiment 1).

The experiment took place in the same dimly lit room as was used in Experiment 1. Participants sat at a desk with a desktop computer in front of them consisting of a mouse, keyboard, and a 17″ cathode ray tube screen running at 100 fps. Observers sat approximately 60 cm from the screen and kept their eyes level with the screen by adjusting the chair once before starting the experiment.

Upon completing the task, participants completed the positive mood induction task for 3 minutes. Participants were then verbally debriefed and also given a debriefing form. Overall, testing lasted approximately 40 min.

#### 4.3.6 Data Analyses

Our measure for the facing-the-viewer bias was based on modelling the collected observer responses in terms of the same generalized linear model (GLM) that was described in Experiment 1. Using this model, we derived a measure of the facing-the-viewer bias. We will henceforth refer to this dependent variable as FTV scores.

We used 3 (Condition) ×p3 (Stimulus) mixed design ANOVAs to analyze FTV scores and reaction times. All methods related to post-hoc comparisons, corrections for multiple comparisons, and use of Wilk’s λ were the same as in Experiment 1.

## Results

### Sample Statistics

Participants’ mean state anxiety score from the STAI was 29.56 (*SD* = 5.64), mean trait anxiety score from the STAI was 35.95 (*SD* = 7.52), and mean social interaction anxiety score from the SIAS was 18.73 (*SD* = 8.82). All three outcomes were nearly identical to those found in Experiment 1, and are typical of nonclinical populations, indicating normal levels of anxiety symptoms. To examine whether there were any gender differences in our data, we used individual sample *t* tests to compare men and women. Men and women did not differ significantly in terms of age, heart rate (before or after exercise), any anxiety measure, or FTV scores for any of the levels of factor Stimulus (all *p*’s>.05).

### Number of Missed Responses

For the perceptual task data, a 3 (Exercise) ×3 (Stimulus) mixed design ANOVA on the number of missed trials (i.e., no response within 2 s of previous stimulus presentation) revealed no effect of Exercise (*F*<1), no effect of Stimulus (*F*<1), and no significant interaction (*F*<1). On average, participants missed 2.51% (i.e., about 4 trials) of the walker stimuli (*SD* = 2.40%).

### Heart Rate

We performed a one-way ANOVA on the difference between pre- and post-manipulation heart rates across levels of Exercise. Heart rates were measured in beats per minute (bpm). One participant (a female in the relaxation condition) was not included in these analyses as her post-manipulation heart rate was not recorded. The ANOVA was significant, *F* (2, 51) = 6.50, *p* = .003, η^2^_partial_ = .21, with participants having significantly greater *decreases* in heart rate (relative to baseline) in the relaxation condition (*M* = −4.71 bpm, *SD* = 8.15) compared to in the anxiety induction condition (*M* = 3.37 bpm, *SD* = 7.45, *p* = .002), where heart rates actually increased compared to baseline. Neither the relaxation condition (*p* = .243) nor the anxiety induction condition (*p* = .220), however, differed significantly from the control group (*M* = −0.67 bpm, *SD* = 3.69) in terms of differences in heart rate before and after the anxiety manipulation. In addition, we ran separate one-sample *t* tests to compare the differences in heart rate to zero for each condition. We found of the three conditions, only the relaxation condition differed significantly, with heart rates significantly decreasing relative to baseline measures, *t*(16) = −2.38, *p* = .030. Participants’ heart rates did not differ significantly relative to baseline in either the anxiety induction condition (*p* = .064) or the control group (*p* = .454).

### Visual Analogue Anxiety Measure

We performed a one-way ANOVA on the difference between pre- and post-exercise visual analogue anxiety scores across levels of Condition. Anxiety was measured (in cm) from right to left (with greater values indicating greater anxiety). The ANOVA was significant, *F* (2, 52)  = 11.00, *p*<.001, η^2^_partial_ = .30, with participants having significantly greater *increases* in anxiety (relative to baseline) in the anxiety induction condition (*M* = 0.55 cm, *SD* = 0.18) compared to in either the relaxation condition (*M* = −0.60 cm, *SD* = 0.18, *p*<.001) or the control condition (*M* = −0.26 cm, *SD* = 0.18, *p* = .007). There was no difference in anxiety, however, between the relaxation and control conditions (*p* = .580). Of note, this latter null finding may have been due to floor effects, as the mean anxiety scores on this task measured immediately after the anxiety manipulation were just 0.67 cm (control group) and 0.56 cm (relaxation group). As we did with differences in heart rate, we ran separate one-sample *t* tests to compare differences in anxiety to zero for each condition. We found that anxiety increased significantly compared to baseline in the anxiety induction condition, *t*(18) = 2.17, *p* = .044, but decreased significantly in both the control, *t*(17) = −2.77, *p* = .013, and relaxation conditions, *t*(17) = −4.30, *p*<.001.

### FTV Scores

We analyzed effects on FTV scores with a 3 (Condition) ×3 (Stimulus) mixed design ANOVA. We found both a significant main effect of Stimulus, Wilk’s λ = .36, *F* (2, 104) = 44.34, *p*<.001, η^2^_partial_ = .64, and Condition, *F* (2, 52) = 2.14, *p* = .045, η^2^_partial_ = .11. The Condition x Stimulus interaction, however, was not significant, Wilk’s λ = .92, *F* (4, 104) = 1.07, *p* = .374, η^2^_partial_ = .04.

**Figure 4:**
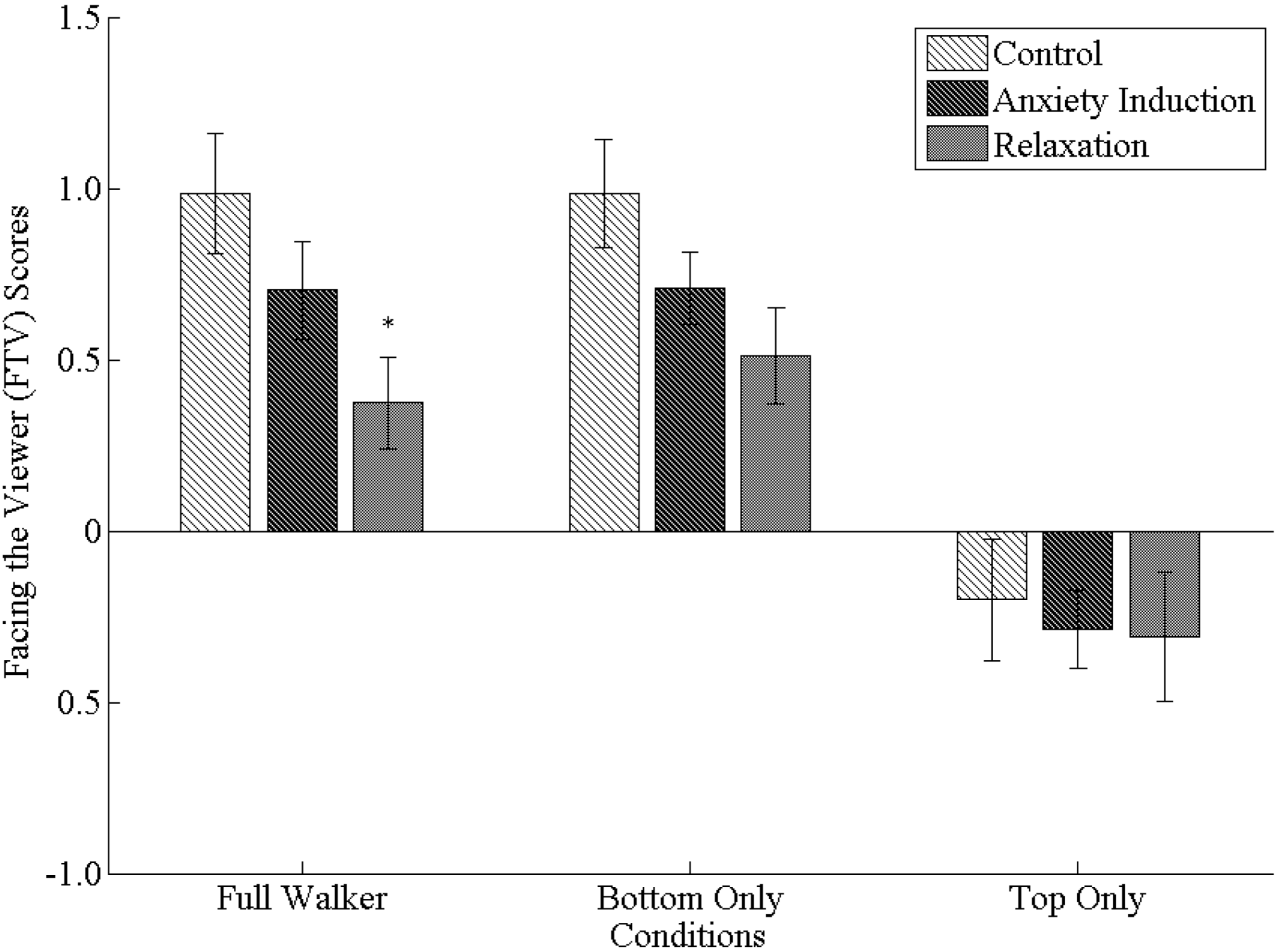
Mean facing-the-viewer (FTV) scores for full stick figure walkers, bottom-half-only stick figure walkers, and top-half-only stick figure walkers (grouped on x-axis) in Experiment 2. Mean FTV scores are displayed separately for the control, anxiety induction, and relaxation conditions (grouped as differently shaded bars). Note that *indicates a significant simple main effect, whereby FTV scores in the relaxation condition were significantly lower than either the walking or jogging conditions for full stick figure walkers only. No other simple main effects were significant. Error bars represent standard error of the mean.

For the significant main effect of Stimulus, post-hoc comparisons revealed that FTV scores for top-half-only walkers (*M* = −0.27, *SD* = 0.09) were significantly lower than for either full (*M* = 0.69, *SD* = 0.09, *p*<.001) or bottom-half-only stick figure walkers (*M* = 0.73, *SD* = 0.08, *p*<.001). Full and bottom-half-only stick figure walkers, however, did not differ significantly from each other. To further examine these biases, we performed one-sample *t* tests on FTV scores separately for each level of Stimulus (collapsed across all levels of Condition) against *b* = 0 (i.e., no bias). These *t* tests revealed that both full and bottom-half-only stick figures elicited significant facing-the-viewer biases, while top-half-only stimuli elicited a significant facing away bias (i.e., a negative FTV score; all cases *p*<.001; see Figure 4). This was the same pattern of results that we observed in Experiment 1.

For the significant main effect of Condition, post-hoc comparisons revealed that FTV scores were significantly lower (across all stimulus types) in the relaxation condition (*M* = 0.19, *SD* = 0.11) than in the control (*M* = 0.59, *SD* = 0.11, *p* = .040). There were no differences, however, between the relaxation condition and the anxiety induction condition (*M* = 0.38, *SD* = 0.11, *p* = .714), or the anxiety induction condition and the control (*p* = .502).

Despite not finding a significant Condition x Stimulus interaction, we conducted a simple main effects analysis on the data (i.e., looking at all possible comparisons between each level of Stimulus separately for each level of Condition) as this information was central to our a priori hypotheses. As hypothesized, we found that participants in the relaxation condition had significantly lower FTV scores than in the control condition (*p* = .020) for full stick figure walkers only (see Figure 4). No other simple main effects comparisons were significant. Although it appears as if FTV scores in the relaxation condition might be lower than in the control condition for bottom-half-only stimuli as well (i.e., upon visual inspection of Figure 4), this difference only approached significance (*p* = .054).

We then performed nine separate one-sample *t* tests for each combination of our two independent variables in order to compare FTV scores with zero (i.e., no bias). For the control condition, we found that while the full and bottom-half-only stick figure walkers elicited significant facing-the-viewer biases (both *p*<.001), FTV scores for top-half-only stimuli (*p* = .277) did not differ significantly from zero (see Figure 4). For the anxiety induction condition, however, we found significant facing-the-viewer biases for both full and bottom-half-only stimuli (both *p*<.001), and a significant facing away bias for top-half-only stimuli (*p* = .023). For the relaxation condition, we once again observed significant facing-the-viewer biases for full (*p* = .013) and bottom-half-only stimuli (*p* = .002), while FTV scores for top-half-only stimuli did not differ significantly from zero (*p* = .120).

### Reaction Time

We examined differences in reaction times (s) on the perceptual task using a 3 (Condition) ×3 (Stimulus) mixed design ANOVA. Contrary to what we observed in Experiment 1, the main effect of Stimulus was significant, Wilk’s λ = .60, *F* (2, 104)  = 16.96, *p*<.001, η^2^_partial_ = .40, as reaction times for full walkers (*M* = 0.66 s, *SD* = 0.03) were significantly shorter than for either bottom-half-only (*M* = 0.69 s, *SD* = 0.03, *p* = .007) or top-half-only stimuli (*M* = 0.72 s, *SD* = 0.03, *p*<.001). Reaction times for bottom-half-only and top-half-only stimuli, however, did not differ significantly (*p* = .075). Neither the main effect of Condition, *F*<1, nor the Condition x Stimulus interaction, Wilk’s λ = .89, *F* (4, 104) = 1.55, *p* = .192, η^2^_partial_ = .06, were significant.

### Correlations

We analysed correlations between anxiety (4 measures: STAI-state, STAI-trait, SIAS, and visual analogue anxiety pre-post difference) and FTV scores for full stick figure walkers separately for each level of Condition, for a total of 45 comparisons (i.e., 15 comparisons per level of Condition). We found no significant correlations in any of the three Exercise conditions (all *p*_adjusted_>.05).

In addition to anxiety, we examined correlations between heart rate measures and FTV scores for full stick figure walkers. We observed no significant correlations between these measures (either in general or separately for each level of Condition).

Lastly, we examined the correlations between reaction times and FTV scores collapsed across all levels of Condition. Unlike in Experiment 1, no correlations were significant after controlling for multiple comparisons.

## General Discussion

The purpose of this study was to test a hypothesis that arises from the theory that the facing-the-viewer bias for biological motion stimuli has a sociobiological cause: Mistaking an approaching human as retreating when he/she is actually approaching is potentially more costly than making the opposite mistake. Given this, we expected that anxious individuals would demonstrate larger facing-the-viewer biases than less anxious individuals since more anxious individuals display a bias towards perceiving more threatening percepts when faced with visual ambiguity [14]–[16].

In the present study, we attempted to manipulate participants’ anxiety indirectly by means of physical exercise (Experiment 1) and by means of inducing or reducing anxiety more directly (Experiment 2). In Experiment 1, we asked participants to perform physical exercise on a treadmill before measuring their facing-the-viewer bias in response to rotating stick figure walkers that differed in terms of degree of sociobiological relevance. As exercise reduces anxiety, we expected that participants would show weaker facing-the-viewer biases after walking or jogging on the treadmill compared to when not exercising (i.e., standing still on a motionless treadmill). Furthermore, as full stick figure walkers have more sociobiological relevance than either bottom-half-only or top-half-only stimuli, we predicted that exercise would only weaken facing-the-viewer biases for full stick figure walkers. As hypothesized, we found that facing-the-viewer biases for full stick figure walkers were significantly weaker in participants who either walked or jogged compared to those in the standing condition, but that physical exercise had no measurable effect on facing-the-viewer biases for either bottom-half-only or top-half-only stimuli.

In Experiment 2, we asked participants to perform a task that was designed to make them either more anxious (anxiety induction task) or less anxious (progressive muscle relaxation), and then complete the exact same perceptual task as was used in Experiment 1. As hypothesized, we found that facing-the-viewer biases for full stick figure walkers were significantly lower for the relaxation condition, but that relaxation did not significantly affect biases for either bottom-half-only or top-half-only stimuli. Participants in the anxiety induction task condition, however, did not differ significantly from those in the control condition in terms of their facing-the-viewer biases, and this was not as hypothesized.

Our hypothesis that exercise would reduce the facing-the-viewer bias was based on the premise that anxiety and the facing-the-viewer bias are positively correlated. The anxiolytic benefits of physical exercise have been shown repeatedly [22]–[27], and since we assessed anxiety before participants completed the treadmill task, we hypothesized that measures of anxiety and the facing-the-viewer bias would only correlate in the standing condition. Although we found no significant correlations when using a strict correction for multiple comparisons, we did observe a significant positive correlation between social interaction anxiety and FTV scores when applying this method less conservatively. This finding suggests that the observed effect of exercise on the facing-the-viewer bias may have indeed been mediated by the anxiolytic properties of physical exercise as we proposed, and replicates the previous finding of a positive correlation between anxiety and the facing-the-viewer bias [20]. In Experiment 2, we sought to account for a methodological problem present in Experiment 1, by adding in an additional measure of anxiety directly after the anxiety manipulation. While we found that participants in the progressive muscle relaxation group had significantly decreases in anxiety (relative to baseline) as well as concomitant reductions in facing-the-viewer biases, we did not find any significant correlations between anxiety and facing-the-viewer biases in Experiment 2. Even so, these results, in accordance with those found in Experiment 1, provide strong evidence of a link between anxiety and facing-the-viewer biases.

One finding that did not support our hypotheses was that the anxiety induction task did not significantly impact facing-the-viewer biases. We designed this imagery task based on similar tasks in the literature that had been successful in increasing subjective anxiety [36]–[38]. Although we observed that subjective anxiety (as measured with a visual analogue scale) increased significantly after partaking in the anxiety induction condition, we did not observe any concomitant changes in facing-the-viewer biases. Future research is needed in order to assess whether our findings are due to inadequate anxiety induction, or whether this is a true null finding.

The significant effect of Stimulus on facing-the-viewer biases that we observed (in both Experiments 1 and 2) also replicates previous findings [6]. Specifically, we found that both full and bottom-half-only stick figure walkers elicited facing-the-viewer biases, while top-half-only stimuli elicited facing away biases. This may occur because local motion features in the lower half of point-light figures (i.e., most notably, the feet) are quickly identified and help to adequately detect biological motion (see [8]). Top-half-only stimuli, on the other hand, are less likely to be readily identified as representing human figures. Alternatively, these differences in facing-the-viewer biases might be related to the convexity and concavity represented in the positioning of the lower or upper limbs (Weech & Troje, unpublished manuscript). Weech and Troje argue that the facing the viewer bias is sensitive to lower level stimulus features such as convexity and concavity, which our visual system already has prior assumptions about (i.e., a bias to perceive convexity rather than concavity when surface normals are ambiguous; [43]). According to Weech and Troje, the upper half of a human biological motion figure in fronto-parallel projection elicits a facing away bias because we assume that we look onto the bended elbows from their convex side. Likewise, the lower part elicits a facing-towards percept because we assume that we look at the knees from their convex side. Weech and Troje’s findings highlight the importance of bottom-up processes in producing the facing the viewer bias. Top-down processes like the one we discuss in the present study clearly do not account for all of the variance in facing biases, nor do we argue such here.

While the results of this study were as we expected, our findings contradict some previous reports regarding the facing-the-viewer bias. For example, while we saw a decrease in facing-the-viewer biases following physical exercise on a treadmill, others have found that walking on a treadmill actually increased facing-the-viewer biases for some point-light figures if the stimuli were displayed performing the same action as the observer [44]. In the latter study, however, perceptual biases were assessed while participants were actually engaged in walking on the treadmill; a key difference from the present study.

Our results also contradict a recent finding that individuals with high social anxiety had significantly weaker facing-the-viewer biases than individuals with low social anxiety [21]. In Experiment 1, we observed that higher social interaction anxiety was associated with greater facing-the-viewer biases (in the standing/control condition). Further research is needed in order to discern why our results differ from this previous finding.

Of interest, in Experiment 1, we unexpectedly observed that greater reaction times were associated with greater facing-the-viewer biases for bottom-half-only stimuli and the opposite trend (i.e., stronger facing away biases) for top-half-only stimuli. On the other hand, we found that reaction times were not related to facing-the-viewer biases for full stick figure walkers. In one previous study, shorter reaction times were associated with *greater* facing-the-viewer biases for full walkers when no time limit was imposed [45]. The authors of that study argued that strong facing-the-viewer biases help to disambiguate a figure, thus allowing its facing direction to be resolved more quickly. We, on the other hand, found the opposite relationship for top-half- and bottom-half-only biological motion stimuli: Stronger biases were associated with shorter reaction times. Note, however, that this finding was not replicated in Experiment 2. Furthermore, contrary to what we observed in Experiment 1, we found that reaction times for full walkers were significantly shorter than for either bottom-half-only or top-half-only stimuli in Experiment 2. Future research on the facing-the-viewer bias will have to carefully consider the amount of time that is given for participants to respond. Such studies will also have to recruit greater sample sizes that are more amenable to correlational statistics than those of Experiments 1 and 2.

One particular difference between the results of Experiments 1 and 2 was the main effect of Condition that we observed in the second experiment, such that facing-the-viewer biases were significantly lower (across all stimulus types) in the relaxation condition compared to the control condition. At first glance, this finding appears to contradict our observations (in both Experiments 1 and 2) that decreases in facing-the-viewer biases purportedly due to anxiety occur only for full stick-figure walkers. We had originally hypothesized that this would be the case because full stick figure walkers hold more social relevance than either bottom-half-only or top-half-only stimuli. Note, though, that the main effect observed in Experiment 2 appears to be driven by the fact that facing-the-viewer biases were almost significantly lower in the relaxation condition (compared to controls) for bottom-half-only stick figure walkers (*p* = .054; see Figure S4). Even if this difference were significant, however, this would not necessarily contradict our assertion that more anxious individuals have greater facing-the-viewer biases because the facing-towards percept of biological motion stimuli is more threatening. For instance, researchers have already noted in the past that our visual system is especially attuned to local features in the bottom half of point light walkers during biological motion perception [8].

The effects of physical exercise and progressive muscle relaxation on perceptual biases that we observed in this study are important. A number of studies have found evidence that anxiety affects attentional [11]–[13] and perceptual biases [13]–[16] towards visual stimuli that are potentially threatening, making it more likely that anxious individuals will perceive threat in their environment. It has been argued that this bias towards the environment may exacerbate anxiety disorders, making them more difficult to treat and thus helping to perpetuate them [11], [17], [18], [46]. Furthermore, experimentally modifying perceptual threat biases in anxious individuals has been found to reduce both measures of threat bias as well as symptoms of anxiety [47]. Our study is the first to demonstrate that physical exercise and progressive muscle relaxation can reduce perceptual biases related to the perception of threat, providing further empirical evidence of the anxiolytic benefits of both of these activities as well as offering another potential mechanism for how these benefits occur: A reduction in threat bias.

## Conclusion

This study is the first to attempt to manipulate anxiety in order to investigate its effect on the facing-the-viewer bias. Multiple studies have confirmed the importance of both high-level social factors and low-level perceptual features in contributing to this perceptual bias. Our study reaffirms the importance of high-level social cognition in contributing to it, and provides empirical evidence that anxiety is positively correlated with it.

## Supporting Information

Table S1 **Note: Values represent Pearson’s r correlation coefficients.** Though not shown, p values were adjusted according to the multiple comparison method outlined by Benjamini and Hochberg (1995) for 9 comparisons and adjusted p values were then compared with α = .05. *indicates significant at α<.05 level, **indicates significant at α<.01 level.
(TIF)

Video S1 **Example trials from the perceptual task (used in both Experiments 1 and 2) depicting full, bottom-half-only, and top-half-only walkers.** Despite that fact that all walkers were rendered rotating counterclockwise, observers can perceive them rotating in either direction.
(AVI)
