# Supplementary figures and images for: Both Physical Exercise and Progressive Muscle Relaxation Reduce the Facing-the-Viewer Bias in Biological Motion Perception
Source: PLoS One. 2014 Jul 2;9(7):e99902. doi: 10.1371/journal.pone.0099902 (PMC4079562; doi:10.1371/journal.pone.0099902)

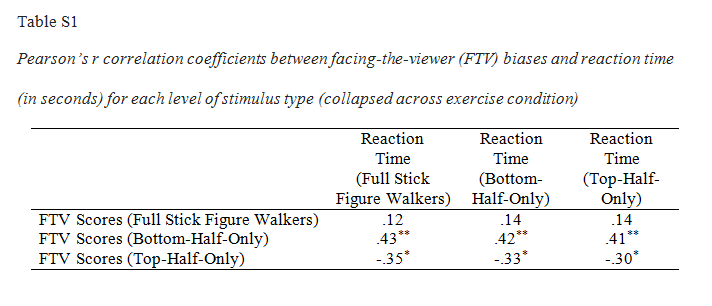

Supplement: Table S1 — Note: Values represent Pearson’s r correlation coefficients. Though not shown, p values were adjusted according to the multiple comparison method outlined by Benjamini and Hochberg (1995) for 9 comparisons and adjusted p values were then compared with α = .05. *indicates significant at α<.05 level, **indicates significant at α<.01 level. (TIF) [file pone.0099902.s001.tif]
